# MAFG-DT promotes prostate cancer bone metastasis through activation of the Wnt/β-catenin pathway

**DOI:** 10.3389/fonc.2024.1461546

**Published:** 2024-12-13

**Authors:** Chongwen Wang, Zheng Zhou, Yongjie Ye, Liqiang Zhou, Jialun Wang, Zhi Zhang

**Affiliations:** Department of Orthopedics, Chengdu Fifth People’s Hospital, Chengdu, China

**Keywords:** MAFG-DT, prostate cancer, Wnt/β-Catenin pathway, miR-24-3p, FZD4, FZD5

## Abstract

**Background:**

Prostate cancer (PCa) ranks as the second leading cause of cancer-related mortality among men. Long non-coding RNAs (lncRNAs) are known to play a regulatory role in the development of various human cancers. LncRNA MAFG-divergent transcript (MAFG-DT) was reported to play a crucial role in tumor progression of multiple human cancers, such as pancreatic cancer, colorectal cancer, bladder cancer, and gastric cancer. Nevertheless, the specific function of MAFG-DT in the context of bone metastasis in PCa remains inadequately understood.

**Methods:**

The expression level of MAFG-DT was analyzed in published datasets and further confirmed in clinical samples and cell lines by RT-qPCR and *in situ* hybridization assays. Additionally, we further examined the effect of MAFG-DT on cell proliferation, migration, invasion and bone metastasis through CCK8, EdU, colony formation, transwell assays and bone metastasis model with intracardiac injection. Subsequently, the specific mechanism of MAFG-DT in PCa was investigated by RIP, ChIP, bioinformatic analysis and luciferase reporter assays.

**Results:**

We found that MAFG-DT expression was significantly upregulated in PCa tissues exhibiting bone metastasis. Elevated levels of MAFG-DT expression were found to be positively associated with poor prognostic outcomes in PCa patients. Functionally, the knockdown of MAFG-DT resulted in a pronounced inhibition of cellular proliferation, migration, invasion, and bone metastasis. Moreover, it was demonstrated that MAFG-DT enhanced the expression of FZD4 and FZD5 mRNAs by sequestering miR-24-3p, thereby activating the Wnt/β-catenin signaling pathway. Additionally, the transcription factor MAFG was found to transcriptionally activate MAFG-DT in PCa.

**Conclusion:**

This study confirms the oncogenic role of MAFG/MAFG-DT/miR-24-3p/Wnt/β-catenin in PCa, which suggests that MAFG-DT could serve as a potential therapeutic target for PCa.

## Introduction

Prostate cancer (PCa) is the second leading cause of cancer-associated mortality among men (1–3). Given the improvements that have been made in terms of the diagnosis of PCa and its treatment methods, the survival rate of patients with PCa has improved over the course of the last decade (2, 4, 5). Inhibition of the androgen receptor is the classical therapeutic method for PCa patients, and patients benefit from this therapy in the early stages of the disease (6). However, over time, a majority of patients will develop resistance to androgen deprivation therapy. Patients with advanced PCa with metastasis have a poor quality of life and increased rates of mortality. Therefore, gaining an understanding of the underlying molecular mechanism that is responsible for PCa progression will contribute towards our ability to control PCa.

LncRNAs are involved in the regulation of many biological and pathological processes, such as cell migration and invasion (7). LncRNAs are able to interact with DNAs, RNAs, and proteins, thereby participating in the development of multiple types of human cancer (8–11). The dysregulation of lncRNAs has been reported in tumorigenesis, metastasis, drug resistance, and radiation resistance (12–16). The lncRNA MAFG-divergent transcript (MAFG DT) has been shown to be upregulated in pancreatic cancer, colorectal cancer, bladder cancer, and gastric cancer, and it promotes tumor progression (17–20). However, to the best of the authors’ knowledge, the biological and clinical roles of MAFG-DT in PCa bone metastasis remain unclear.

The Wnt/β-catenin pathway has been reported to be dysregulated in numerous diseases, including adolescent idiopathic scoliosis, Alzheimer’**s** disease, and various types of human cancer (21, 22). Wnt/β-catenin signaling often serves an oncogenic role in tumor progression. For example, AID induced the demethylation of CXCL12, which stabilized the Wnt signaling pathway executor β-catenin, further promoting EMT process and PCa metastasis (23). CRNDE was found to enhance the proliferation and chemoresistance of colorectal cancer via activating Wnt signaling through sponging of the miR-181a-5p (24). However, the underlying mechanism of lncRNA-mediated activation of the Wnt/β-catenin signaling in PCa bone metastasis was rarely known.

In this study, it is shown how MAFG-DT is upregulated in PCa tissues, especially in bone metastatic PCa tissues. Increased levels of MAFG-DT were found to be positively correlated with the poor prognosis of patients with PCa. Functionally, MAFG-DT knockdown dramatically inhibited PCa cell proliferation, migration, invasion, and bone metastasis. Mechanistically, MAFG-DT upregulated FZD4 and FZD5 expression via sponging miR-24-3p to abolish the miR-24-3p-mediated degradation of FZD4/5, which further activated the Wnt/β-catenin pathway. Collectively, the findings of the present have provided novel insights into how MAFG-DT promotes PCa progression, suggesting that MAFG-DT may have the potential for development into a putative therapeutic target against PCa.

## Materials and methods

### Human tissues and cell culture

The human tissues used in the present study were collected from patients undergoing surgery in our hospital. The Institutional Research Ethics Committee at the Chengdu Fifth People’s Hospital sanctioned the study protocol for utilizing clinical materials in research (No.2022-CDFH-H-63).Bone metastatic tissues were obtained from spine lesions. Cell culture was performed according to the procedures presented in the **Supplementary Materials**.

### Plasmids, siRNA, miRNA mimics, and inhibitor and transfection

Human MAFG-DT plasmid and two shRNA plasmids against MAFG-DT were purchased from Sangon Biotech (Shanghai, China). The corresponding blank plasmid vectors were used as the negative control. The plasmids were transfected into cells using Lipofectamine 3000 transfection reagent (Invitrogen, USA) based on the manufacturer’s instructions. For the establishment of stable cell lines, shRNA lentiviral plasmids, packaging, and envelop plasmids were transfected into 293T cells to produce the lentivirus. The lentivirus was collected in a 15mL tube and centrifuged (500g/5min) to remove cellular debris and impurities and then filtered using a filter (0.45um). The collected virus supernatant can be used for infection and storage. PCa cells (30%-50% density) were infected by the lentivirus for 3 days and the MOI of infection was 10. siRNAs were used to knock down some genes and obtained from Riobio (China). The shRNA and siRNA sequences were shown in **Supplementary Table 1**. miR-24-3p mimics, inhibitor and corresponding negative controls were purchased from Riobio (Guangzhou, China). The siRNAs, miRNA mimics and inhibitor, and NC were incubated with riboFECT™ CP transfection reagent (Riobio) at room temperature for 15 minutes based on the manufacturer’s instructions.

### Real-time quantitative PCR

RT-qPCR assay was performed precisely following the protocol in a previous study (25). The sequence of primers used in the present study were presented in **Supplementary Table 2**.

### Bone metastasis model

The intracardiac-injection model of bone metastasis was established according to the guidelines provided in a previous study (3). In every group, five mice (BALB\c-nu, male, 4-6 weeks) were injected with PC3 cells (1×10^6^ cells) in a solution of 100μl PBS into the cardiac ventricle. Mice were anesthetized with isoflurane (inhalation anesthesia, 3% for induction, and 1.5% for maintenance), and at the end of the experiment, the mice were euthanized with CO_2_ inhalation (CO_2_ replacement rate, 30%). The criteria for death were as follows: i) the animals had no heartbeat for more than 5 minutes; ii) corneal and nerve reflexes disappeared, and the pupils of the mice were dilated. The osteolytic score based on X-ray analysis was calculated, consistently with the method outlined in a previously published study (3). All animal experiments were approved by the Institutional Animal Care and Use Committee of the Chengdu Fifth People’s Hospital (No. 2022-CDFH-A-19).

### Western blotting

Western blotting was conducted with the standard method. Antibodies against FZD4 (ab277797, 1:500), FZD5 (ab75234, 1:1000) and β-catenin (ab32572, 1:3000) were obtained from Abcam, and E-cadherin (20874-1-AP, 1:3000) and Vimentin (10366-1-AP, 1:2000) were obtained from Proteintech. GAPDH (60004-1-Ig, 1:50000, Proteintech) were utilized as the protein loading controls. The detailed protocol of the Western blotting assay was presented in **Supplementary Materials**.

### Statistical analysis

Data analysis was conducted using GraphPad Prism 7.0. The results are shown as the mean ± standard deviation. Student’s t-test (paired or unpaired) was used to determine the differences between the two groups. P < 0.05 was considered to indicate a statistically significant difference.

## Results

### The high expression of MAFG-DT is associated with bone metastasis in patients with PCa

**Figure 1:**
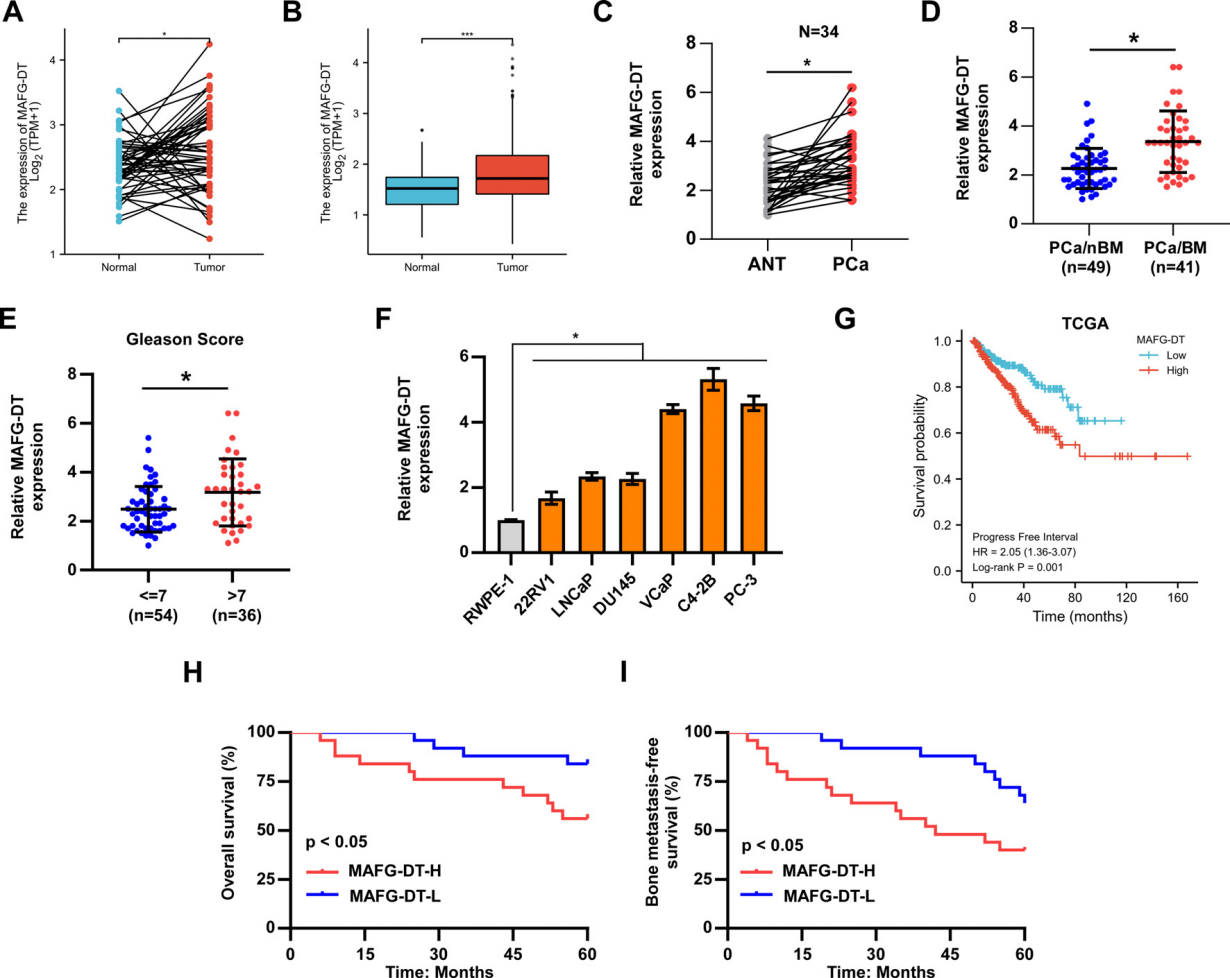
The upregulation of MAFG-DT is related to bone metastasis and poor prognosis in patients with PCa. **(A)** MAFG-DT expression was analyzed in prostate cancer (Tumor, n=52) and paired adjacent normal tissue (Normal, n=52) from TCGA dataset. **(B)** MAFG-DT expression was analyzed in prostate cancer (Tumor, n=499) and adjacent normal tissue (Normal, n=52) from TCGA dataset. **(C)** Real-time qPCR analysis was used to determine MAFG-DT expression in PCa (n=34) and paired adjacent normal tissues (ANT, n=34) from our hospital. **(D)** Real-time qPCR analysis was used to determine MAFG-DT expression in PCa tissues without bone metastasis (PCa/nBM) and PCa tissues with bone metastasis (PCa/BM) from our hospital. **(E)** Real-time qPCR analysis was used to determine MAFG-DT expression in PCa tissues (Gleason score <7 or ≥7) from our hospital. **(F)** Real-time qPCR analysis was used to determine MAFG-DT expression in PCa cell lines (22RV1, LNCaP, DU145, VCaP, C4-2B, PC-3) and normal prostate cell (RWPE-1). **(G)** Kaplan–Meier analysis of progression-free survival curve of the PCa patients stratified by MAFG-DT expression in TCGA dataset. **(H, I)** Kaplan–Meier analysis of overall **(H)** and bone metastasis-free **(I)** survival curves of the PCa patients stratified by MAFG-DT expression in our cohort. All experiments were performed in biological triplicate. Statistical analyses were performed by ANOVA test **(f)**, unpaired Student’s t-test **(B, D, E)**, paired Student’s t-test **(A, C)**, and the log-rank test **(G–I)**. *P < 0.05.

MAFG-DT is found to be dysregulated in multiple types of cancer (17, 26, 27); nevertheless, the expression pattern of MAFG-DT in PCa bone metastasis remains unknown. Therefore, the MAFG-DT level in PCa was analyzed from The Cancer Genome Atlas (TCGA) database, and the results indicated that a high level of MAFG-DT was more prevalent in PCa tissues relative to the adjacent normal tissues (**Figures 1A, B**). Next, the MAFG-DT expression in our clinical samples was investigated, and the findings were similar to the results obtained from TCGA (**Figure 1C**). Further analysis found that the MAFG-DT expression was increased in bone metastatic PCa tissues (PCa/BM) compared with non-bone metastatic PCa tissues (PCa/nBM) (**Figure 1D**). Furthermore, the MAFG-DT level was found to be higher in PCa with a Gleason Score ≥7 compared with PCa with a Gleason Score <7 (**Figure 1E**). Subsequently, the expression of MAFG-DT was assessed in PCa cells, and the finding indicated that the level of MAFG-DT was markedly increased in PCa cells, especially in bone-metastatic cells, compared with RWPE-1 cell (normal prostate cell line) (**Figure 1F**). Survival analysis based on TCGA-PCa dataset was then investigated. This analysis indicated that an increased expression of MAFG-DT was positively correlated with poor progression-free survival (**Figure 1G**). In our PCa patient cohort, a high level of MAFG-DT was found to predict shorter overall survival, and bone metastasis-free survival, rates (**Figures 1H, I**). Taken together, these experiments revealed that the level of MAFG-DT is increased in cases of PCa/BM, and that this is one predictive factor of the prognosis for PCa patients.

### MAFG-DT promotes PCa cell proliferation *in vitro*

**Figure 2:**
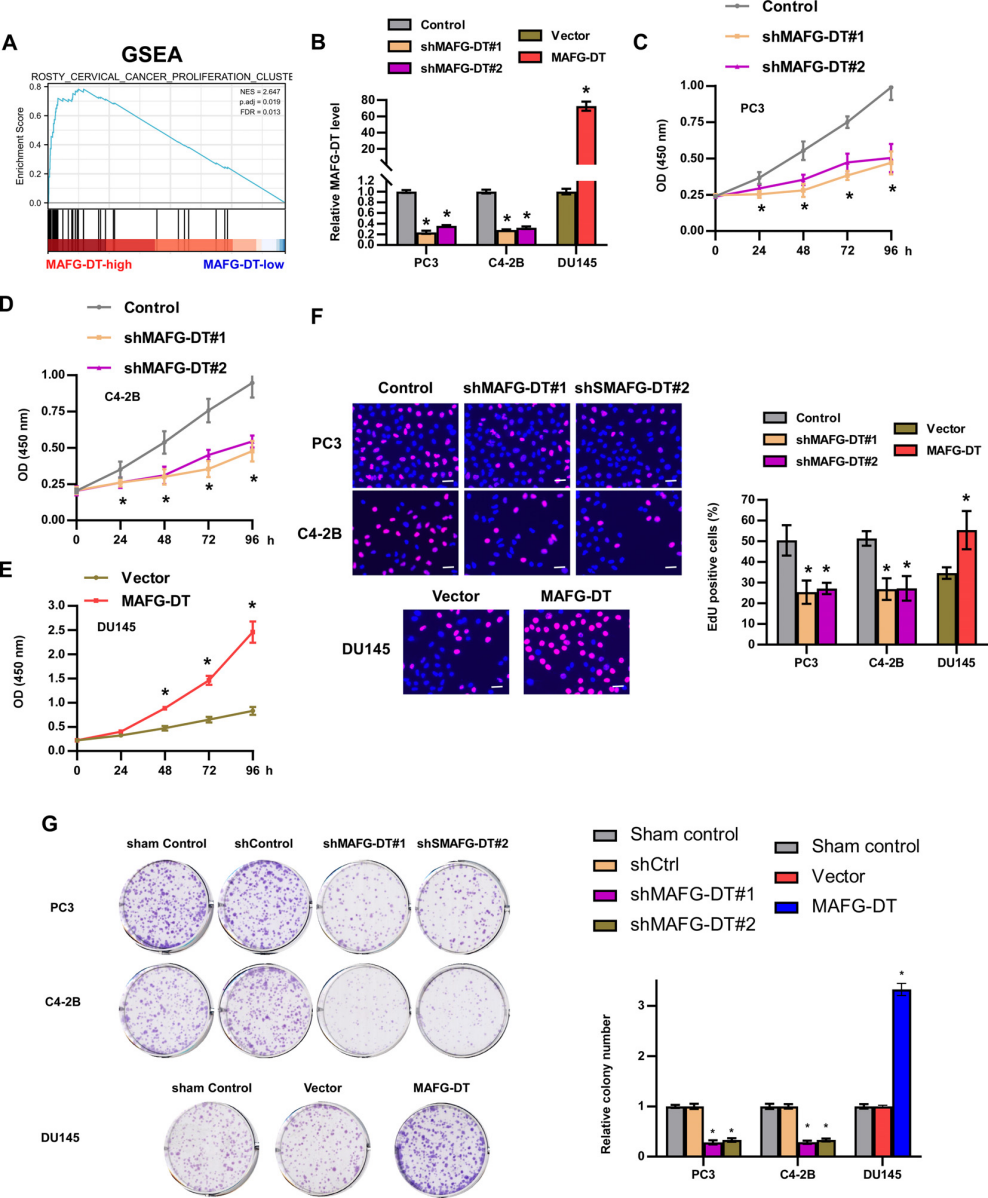
MAFG-DT promotes PCa cell proliferation *in vitro*. **(A)** Gene Set Enrichment Analysis showed that MAFG-DT was related to cell proliferation based on TCGA dataset. **(B)** Real-time qPCR analysis was used to determine MAFG-DT expression to verify the overexpression and knockdown efficiency in the indicated cells. **(C–E)** CCK8 assay was performed to investigate the cell viability in the indicated cells. **(F)** EdU assay was performed to determine the cell proliferation ability in the indicated cells. **(G)** Colony formation assay was conducted to test colony formation ability in the indicated cells (left panels). Histogram analysis of the fold change of colony is shown (right panels). All experiments were performed in biological triplicate. Statistical analyses were performed by ANOVA test **(B–G)** and unpaired Student’s t-test **(B, F, G)**. *P< 0.05.

Based on TCGA data, Gene Set Enrichment Analysis (GSEA) was first performed to examine the biological role of MAFG-DT in the progression of PCa. A high expression level of MAFG-DT was observed to be associated with increased proliferation (**Figure 2A**). Subsequently, experiments were devised wherein MAFG-DT knockdown was performed by using two shRNAs targeting MAFG-DT in PC3 and C4-2B cells, and MAFG-DT was overexpressed in DU145 cells, which were verified using RT-qPCR analysis (**Figure 2B**). The viability of the PC3 and C4-2B cells was found to be decreased upon MAFG-DT inhibition, as shown by CKK8 assay, whereas MAFG-DT overexpression elicited the opposite effects in DU145 cells (**Figures 2C–E**). According to the EdU assay, inhibition of MAFG-DT significantly decreased, whereas MAFG-DT overexpression increased the percentage of EdU^+^ PCa cells (**Figure 2F**). Meanwhile, the number of cell colonies was suppressed in PCa cells with knockdown of MAFG-DT, whereas the number of colonies was enhanced in MAFG-DT-overexpressing PCa cells (**Figure 2G**). Collectively, these results indicate that MAFG-DT could enhance the proliferation ability of PCa cells.

### MAFG-DT promotes PCa cell invasion and migration *in vitro* and bone metastasis *in vivo*

**Figure 3:**
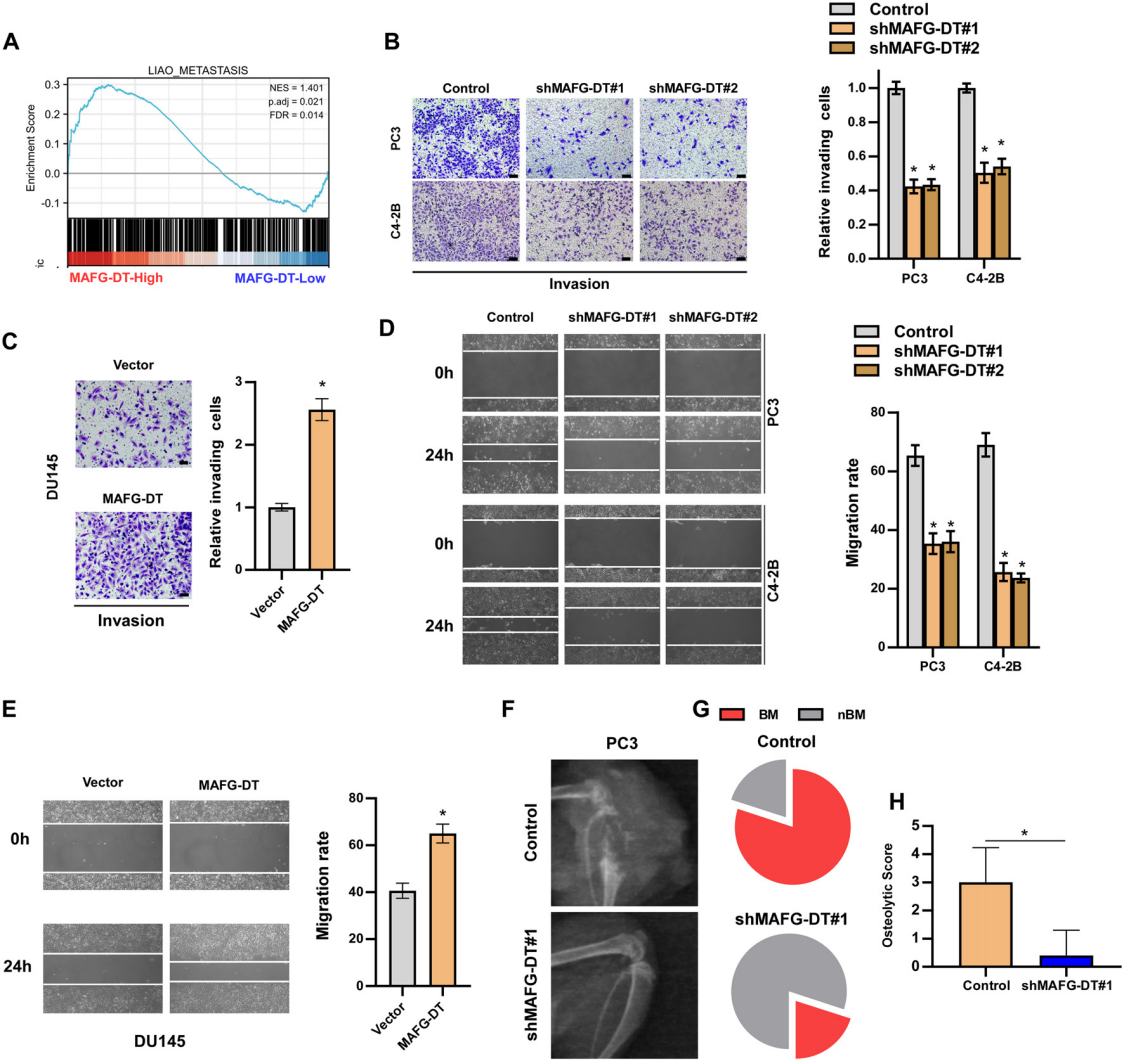
MAFG-DT promotes PCa cell invasion and migration *in vitro*, and bone metastasis *in vivo*. **(A)** Gene Set Enrichment Analysis showed that MAFG-DT was related to tumor metastasis. **(B, C)** A Transwell assay was performed to analyze the invasion of PC3, C4-2B and DU145 cells in the indicated groups (left panels). Histogram analysis of fold change of invasion is shown (right panels). **(D, E)** A wound healing assay was performed to analyze the migration of PC3, C4-2B and DU145 cells in the indicated groups (left panels). Histogram analysis of fold change of migration is shown (right panels). **(F)** The representative X-ray image of mouse hindlimb in the indicated group. **(G)** The incidence of bone metastasis in the indicted group. **(H)** The osteolytic score of mouse hindlimbs based on X-ray in the indicated group. All experiments were performed in biological triplicate. Statistical analyses were performed by ANOVA test **(D, E)** and unpaired Student’s t-test **(F, G, H)**. *P< 0.05.

GSEA in **Figure 2** also demonstrated that MAFG-DT is highly enriched in the gene signature of tumor metastasis (**Figure 3A**). Subsequently, Transwell and wound healing assays were carried out to investigate the effect of MAFG-DT on the metastatic behavior of the PCa cells. As revealed by Transwell invasion assay, the knockdown of MAFG-DT decreased the numbers of invading PCa cells (**Figures 3B, C**). In addition, wound healing assay showed that PCa cells migrated more slowly when MAFG-DT was silenced (**Figures 3D, E**). By contrast, MAFG-DT overexpression was found to enhance the metastatic ability of the PCa cells (**Figures 3F, G**). Since bone metastasis is an important prognostic factor in PCa patients, and the findings shown in **Figure 1** suggested that MAFG-DT is associated with bone metastasis, the function of MAFG-DT in bone metastasis of PCa was then investigated by establishing an intra-cardiac injection model (28). We found that the ability of PCa cells to metastasize to the bone was markedly suppressed by the knockdown of MAFG-DT (**Figures 3F–H**). Considered together, the above results demonstrate that MAFG-DT promotes the bone metastasis of PCa cells.

### MAFG-DT interacts with miR-24-3p

**Figure 4:**
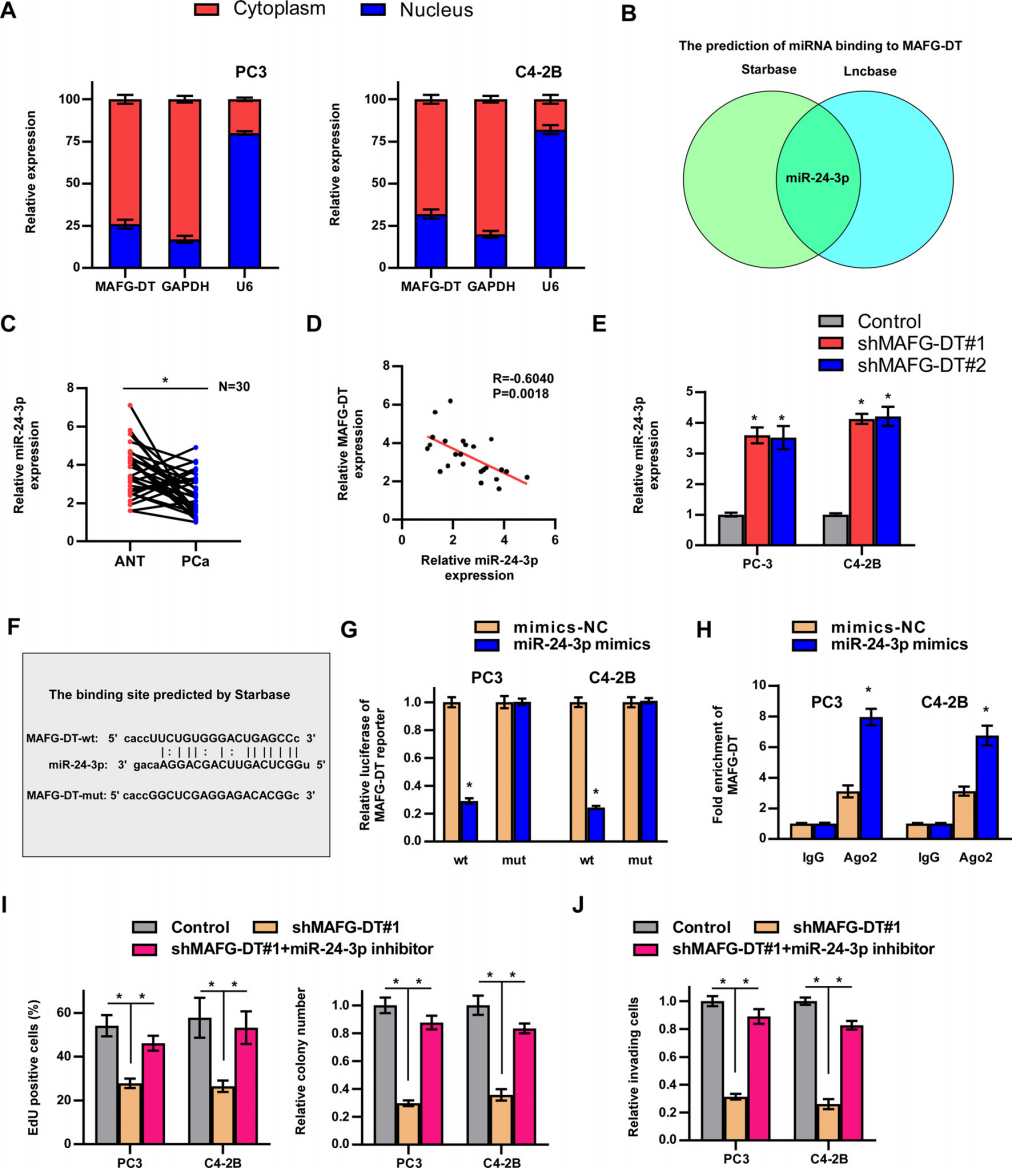
MAFG-DT interacts with miR-24-3p. **(A)** Nuclear/cytoplasmic fractionation analysis was used to detect the subcellular location of MAFG-DT. GAPDH and U6 were used as the controls. **(B)** The prediction of miRNAs that may bind to MAFG-DT by Starbase and Lncbase. **(C)** Real-time qPCR analysis was used to determine miR-24-3p expression in PCa and ANT from our hospital. **(D)** The correlation between MAFG-DT and miR-24-3p level was analyzed in our clinical PCa tissues. **(E)** Real-time qPCR analysis was used to determine miR-24-3p expression in the indicated PC-3 and C4-2B cells. **(F)** The wild-type binding site between MAFG-DT and miR-24-3p was predicted by Starbase and the mutant-type binding site was designed according to the wild-type binding site. **(G)** Luciferase reporter assay showed that MAFG-DT-wt activity was impaired by miR-24-3p in the PC3 and C4-2B cells, whereas MAFG-DT-mut activity remained unchanged. **(H)** RIP assay showed the enrichment of MAFG-DT in Ago2 and IgG protein in the indicated cells. **(I)** EdU (left panels) and colony formation (right panels) assay was conducted to test the effect of miR-24-3p on cell proliferation induced by MAFG-DT in the PC3 and C4-2B cells. **(J)** A Transwell assay was performed to analyze the invasion of PC3 and C4-2B cells in the indicated groups. All experiments were performed in biological triplicate. Statistical analyses were performed by ANOVA test **(E, I, J)**, unpaired Student’s t-test **(G, H)**, paired Student’s t-test **(C)**, and Spearman correlation test **(D)**.*P< 0.05.

There is often a correlation between the expression of antisense lncRNAs and their sense chain genes, suggesting that antisense lncRNAs may be widely involved in the expression regulation of protein coding genes (29). Therefore, we examined the effect of MAFG-DT on the expression of MAFG and the results showed that MAFG-DT could not change the RNA level of MAFG (**Supplementary Figure S1A**). LncRNAs play oncogenic roles dependent on their subcellular localization (1). Subcellular fraction and RT-qPCR assays showed that MAFG-DT was predominantly distributed in the cytoplasm of PCa cells (**Figure 4A**). Cytoplasmic lncRNAs were primarily reported to be competitive endogenous RNAs (ceRNA) to sponge miRNA (30). By analyzing public databases, StarBase and LncBase, miR-24-3p was predicted to target MAFG-DT (**Figure 4B**). The results from the clinical samples indicated that compared with ANT, MAFG-DT was significantly upregulated in PCa (**Figure 4C**). In addition, a correlation study revealed that miR-24-3p showed the negative correlation with MAFG-DT (**Figure 4D**). Further RT-qPCR analyses revealed that miR-24-3p expression was significantly upregulated in cells wherein MAFG-DT was knocked down (**Figure 4E**). These data suggested that MAFG-DT may regulate miR-24-3p expression through a ceRNA-type manner. To explore whether miR-24-3p directly bind to MAFG-DT, the binding site was predicted, and a mutated binding site was designed (**Figure 4F**). Luciferase reporter assay was performed to confirm whether direct binding could occur, and the results showed that the luciferase activity of the MAFG-DT-wt reporter was decreased in PCa cells treated with miR-24-3p mimics, whereas the MAFG-DT-mut reporter’s activity was not significantly changed following the same treatment (**Figure 4G**). RIP assay was then employed to show that treatment with miR-24-3p mimics led to the marked increase in the enrichment of MAFG-DT in argonaute-2 (Ago2) protein (**Figure 4H**), indicating an increased interaction between miR-24-3p and MAFG-DT. To further study whether miR-24-3p mediates the oncogenic function of MAFG-DT in PCa, miR-24-3p inhibitor was found to partly reverse the inhibitory effect of knocking down MAFG-DT on the proliferative and invasive capabilities of the PCa cells (**Figures 4I, J**). Taken together, these results demonstrated that MAFG-DT can promote tumor progression by targeting miR-24-3p.

### MAFG-DT activates Wnt/β-catenin pathway by upregulating FZD4/5 expression

**Figure 5:**
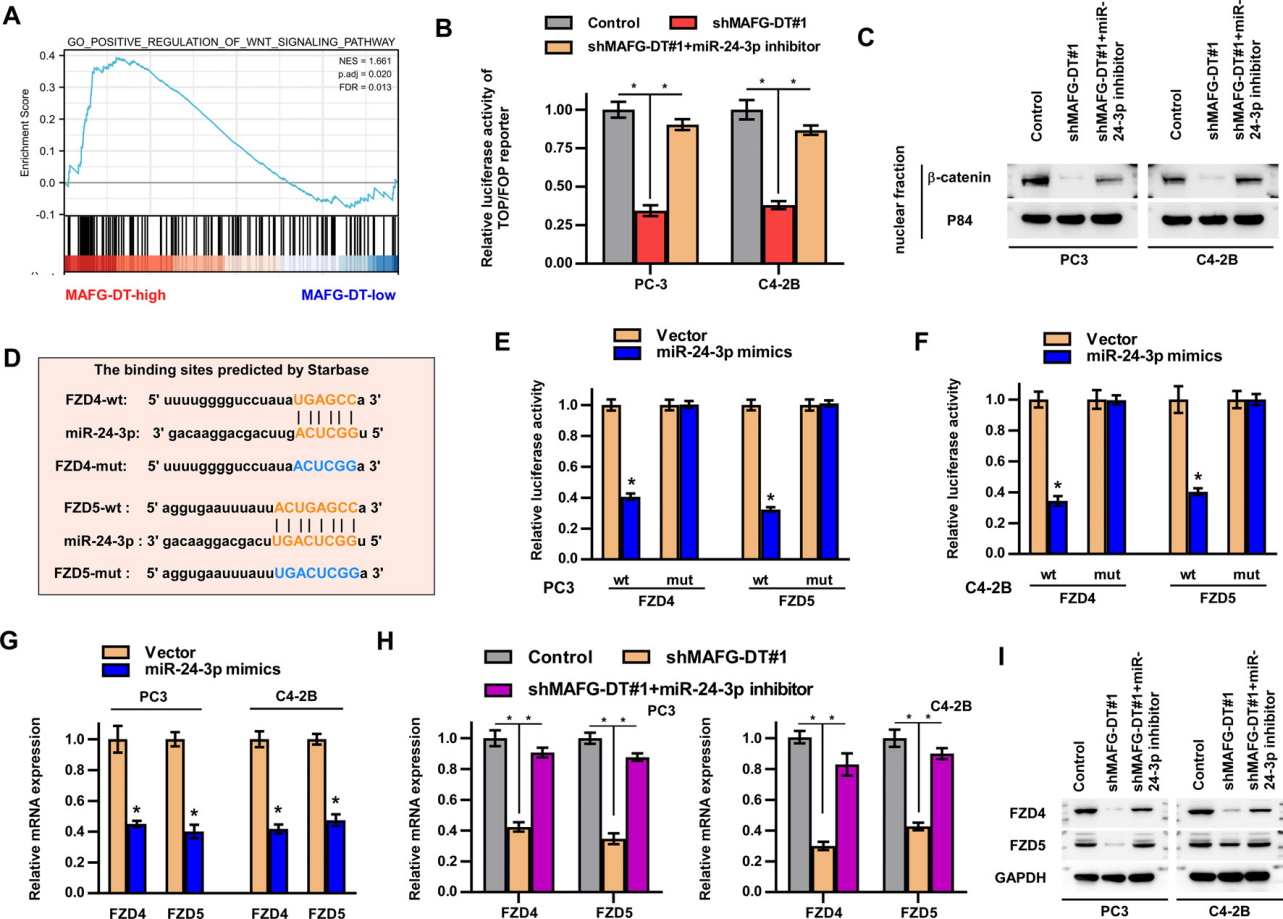
MAFG-DT activates Wnt/β-catenin pathway by upregulating FZD4/5 expression. **(A)** Gene Set Enrichment Analysis showed that MAFG-DT was enriched in the Wnt/β-catenin pathway. **(B)** Luciferase reporter assay was performed to determine the activities of the Wnt/β-catenin pathway in the indicated groups. **(C)** Western blot assay was performed to determine total and nuclear β-catenin protein expression in the indicated groups. GAPDH and P84 were used as the controls. **(D)** The wild-type and mutant-type binding sites between miR-24-3p and FZD4/5 were predicted by Starbase. **(E, F)** Luciferase reporter assay was performed to determine the activities of FZD4/5-wt or -mut reporter in the indicated PC3 and C4-3B cells. **(G, H)** Real-time qPCR analysis was used to determine FZD4/5 levels in the indicated PC3 and C4-3B cells. **(I)** Western blot assay showed the expression of the FZD4/5 proteins in the indicated PC3 and C4-3B cells. All experiments were performed in biological triplicate. Statistical analyses were performed by ANOVA test **(B, H)** and unpaired Student’s t-test **(E-G)**.*P< 0.05.

Signaling pathways have been reported to participate in the development of human cancers (1). To determine which signaling pathways are involved in MAFG-DT-induced tumor progression, GSEA was performed. Wnt/β-catenin signaling was predicted as the downstream pathway of MAFG-DT (**Figure 5A**). Further luciferase reporter and western blotting assays showed that MAFG-DT knockdown led to a marked suppression of Wnt/β-catenin pathway, whereas this function was found to be reversed by miR-24-3p inhibitor (**Figures 5B, C**). Meanwhile, wildtype MAFG-DT significantly activated Wnt/β-catenin pathway, whereas mutant MAFG-DT had no effect on Wnt/β-catenin pathway(**Supplementary Figures S1B, C**). Above results indicated that miR-24-3p mediated MAFG-DT-induced activation of Wnt/β-catenin pathway. Since miRNAs exert their function through targeting mRNAs, potential mRNAs which miR-24-3p targets were screened, and this analysis demonstrated that two key component proteins of Wnt/β-catenin signaling, FZD4 and 5, were the putative targets of miR-24-3p. Subsequently, the binding sites between FZD4/5 and miR-24-3p were predicted using Starbase, and corresponding mutants were designed (**Figure 5D**). The results from luciferase reporter assay revealed that treatment with the miR-24-3p mimics led to a marked decrease in the luciferase activity of the wild-type FZD4/5 (FZD4/5-wt) reporter, whereas the activities of the mutated FZD4/5 reporters were unchanged in PCa cells with miR-24-3p mimics treatment (**Figures 5E, F**). Moreover, RT-qPCR assay demonstrated that the expression levels of FZD4/5 were downregulated in PCa cells of miR-24-3p mimics group (**Figure 5G**). The association between MAFG-DT and FZD4/5 was also investigated, and these experiments showed that the expression levels of FZD4/5 were decreased in MAFG-DT-silenced PCa cells, whereas treatment with miR-24-3p inhibitor led to an attenuation of the suppressive effect induced by MAFG-DT knockdown (**Figures 5H, I**). Taken together, this series of experiments showed that MAFG-DT could upregulate the expression of FZD4/5 to activate the Wnt/β-catenin pathway.

### MAFG transcriptionally upregulates MAFG-DT expression in PCa

**Figure 6:**
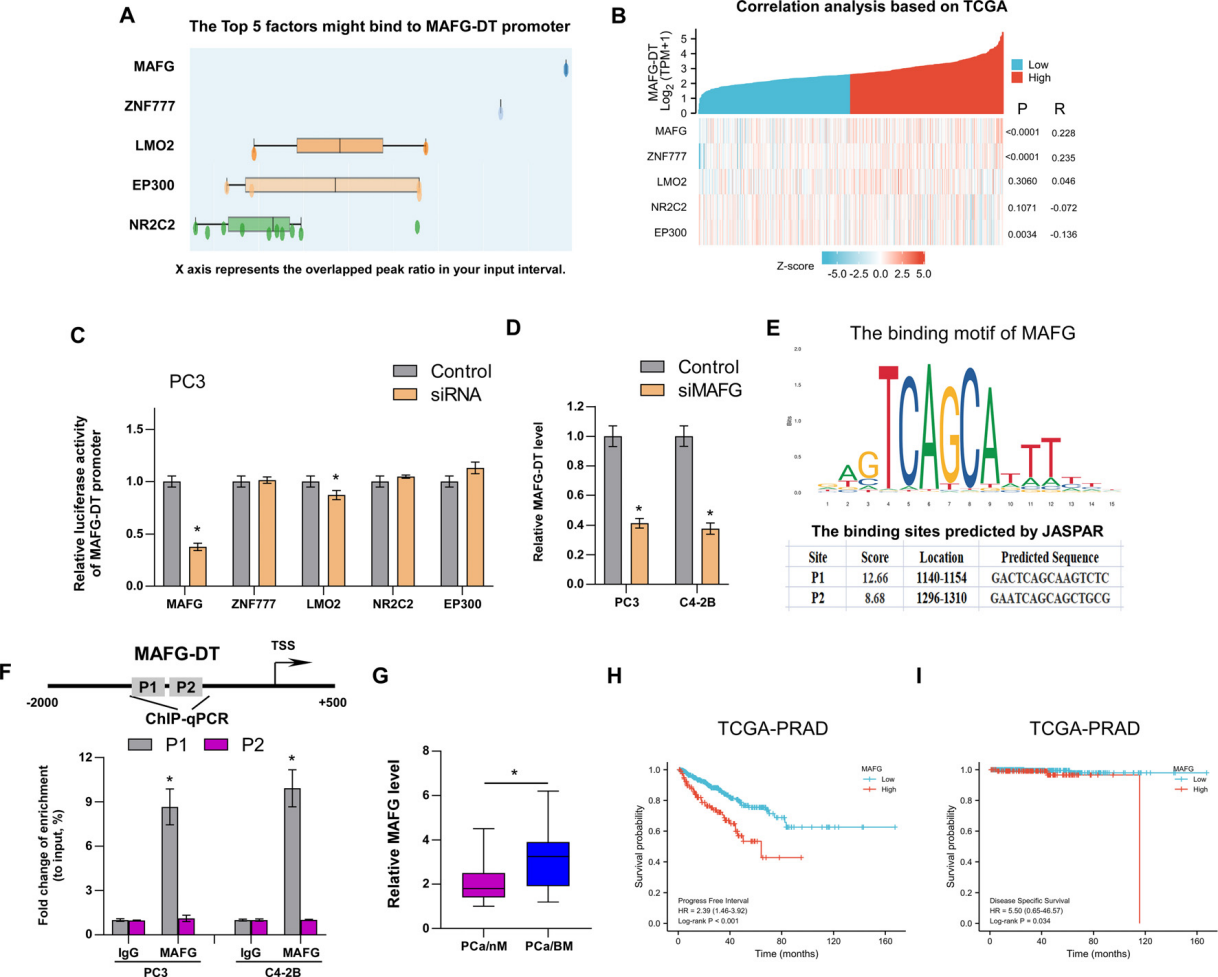
MAFG transcriptionally upregulates MAFG-DT expression in PCa. **(A)** The top 5 factors might bind to MAFG-DT promoter predicted by Cistrome Data Browser. **(B)** The correlation analysis between MAFG, ZNF777, LMO2, NRC2C2, EP300, and MAFG-DT is based on TCGA data. **(C)** The luciferase reporter assay was performed to investigate the effect of MAFG, ZNF777, LMO2, NRC2C2, and EP300 knockdown by siRNAs on the promoter of MAFG-DT in PC3 cells. **(D)** RT-qPCR analysis was used to determine MAFG-DT levels in the indicated PC3 and C4-2B cells transfected with siRNA targeting MAFG. **(D)** The binding motif of MAFG from JASPAR website (upper planes); The binding sites of MAFG on MAFG-DT promoter predicted by JASPAR website (bottom planes). **(E)** ChIP-qPCR analysis was used to verify the predicted binding sites between MAFG and MAFG-DT promoter. **(F)** RT-qPCR analysis was used to determine MAFG levels in PCa/nBM and PCa/BM tissues. **(G, H)** Kaplan–Meier analysis of progression-free **(G)** and disease-specific **(H)** survival curves of the PCa patients stratified by MAFG expression in TCGA cohort. All experiments were performed in biological triplicate. Statistical analyses were performed by unpaired Student’s t-test **(C, D, F, G)**, Spearman correlation test **(B)**, and the log-rank test **(H, I)**. *P < 0.05.

Recently, several studies have demonstrated that transcription factors regulate lncRNA expression (1, 6, 8, 31). To investigate the mechanism of MAFG-DT upregulation in PCa, we analyzed which transcription factors or chromatin factors may be enriched in the promoter of MAFG-DT by using the Cistrome Data Browser (http://dbtoolkit.cistrome.org/). The top 5 factors are shown in **Figure 6A**, and a correlation analysis between these factors and MAFG-DT was performed. The results indicated that MAFG and ZNF777 were positively correlated with MAFG-DT, whereas EP300 had an negative correlation with MAFG-DT (**Figure 6B**). Subsequently, the luciferase reporter assays indicated that silencing MAFG led to a significant decrease in the luciferase activity of the MAFG-DT promoter, exhibiting the biggest fold change (**Figure 6C**). Therefore, MAFG was selected for further investigation, and it was found that MAFG knockdown caused a significant decrease in the expression of MAFG-DT (**Figure 6D**). Subsequently, MAFG was selected for further investigation, and the site of MAFG bound to the MAFG-DT promoter was predicted using the JASPAR website (https://jaspar.genereg.net/; **Figure 6E**). This analysis disclosed the presence of two putative binding sites that MAFG could bind to (**Figure 6E**). ChIP-qPCR assay was then performed to confirm the predicted sites, and the results indicated that it was the P1 site which mediated the binding between MAFG and the MAFG-DT promoter (**Figure 6F**). Finally, we also found that, compared with PCa/nBM, the expression of MAFG was increased in PCa/BM (**Figure 6G**). Survival analysis from TCGA database showed that high MAFG expression predicted poor progression-free and disease-specific survival rates in patients with PCa (**Figures 6H, I**), thereby revealing the clinical significance of MAFG in PCa. Taken together, these results demonstrated that MAFG is able to transcriptionally upregulate MAFG-DT in PCa.

**Figure 7:**
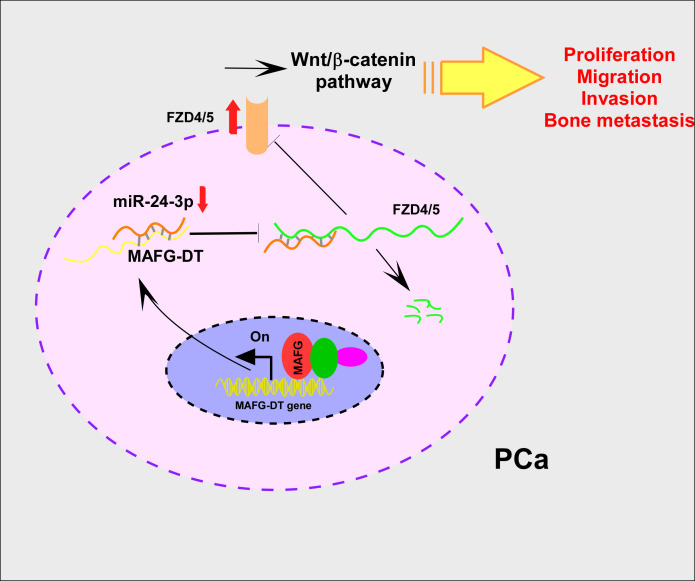
MAFG-DT promotes prostate cancer bone metastasis through activation of the Wnt/β-catenin pathway. Increased MAFG-DT expression was positively correlated with poor overall and bone metastasis-free survivals. Functionally, MAFG-DT knockdown dramatically inhibited the proliferation, migration, invasion and bone metastasis of PCa cells. Mechanically, MAFG-DT upregulated FZD4 and FZD5 expression by sponging miR-24-3p to abolish miR-24-3p-mediated degradation of FZD4/5, which further activated wnt/β-catenin pathway.

Collectively, the results of functional assays revealed that the knockdown of MAFG-DT led to marked suppression of the proliferation, invasion, and migration of PCa cells *in vitro*, and bone metastasis *in vivo*. Mechanistically, MAFG-DT caused the attenuation of miR-24-3p-mediated degradation of FZD4/5, which in turn activated the Wnt/β-catenin signaling pathway. Additionally, transcription factor MAFG upregulated MAFG-DT expression in PCa (**Figure 7**).

## Discussion

Bone metastasis is one of the main characteristics of PCa, contributing to the mortality associated with PCa. However, at present, treatment options for bone metastasis are limited, and it is not possible to prolong the survival rate of patients with PCa. Therefore, improving our understanding of the underlying molecular mechanism responsible for bone metastasis will enable the development of more effective therapies acting against bone-metastatic PCa. This study reveals a novel insight of the ongenic role of MAFG/MAFG-DT/miR 24 3p/Wnt/β-catenin axis in PCa bone metastasis, providing one potential therapeutic target against PCa bone metastasis.

LncRNAs have been shown to be dysregulated in multiple types of human cancer, including gastric cancer, liver cancer, PCa, and pancreatic cancer (32–39). MAFG-DT has been reported to be involved in the progression of a wide variety of different types of cancer (17, 19, 26, 27, 40). For example, MAFG-DT enhanced pancreatic cancer cell proliferation and invasion, whereas it inhibited apoptosis by sponging miR-3196 to upregulate the expression of NFIX (17). In bladder cancer, MAFG-DT was significantly upregulated, and this was associated with advanced clinical and pathological features, which promoted bladder cancer progression by regulating the miR-143-3p/COX-2 signaling axis (19). Furthermore, MAFG-DT upregulated HOXB8 via sponging miR-149-3p to promote colorectal cancer progression (18). However, the biological role and function of MAFG-DT remain unclear in PCa bone metastasis. Therefore, we firstly investigated the expression pattern of MAFG-DT in PCa. Our results indicated that MAFG-DT was upregulated in PCa tissues with bone metastasis relative to PCa tissues without bone metastasis and adjacent normal tissues. Meanwhile, MAFG-DT showed an increased expression in bone-metastatic PCa cell lines compared with other PCa cell lines. Additionally, enhanced MAFG-DT expression was correlated with advanced clinicopathological characteristics and poor prognosis of patients with PCa. These results indicate the oncogenic role of MAFG-DT in PCa bone metastasis. Hence, *in vitro* and vivo experiments were performed to investigate the specific function and mechanism of MAFG-DT in PCa bone metastasis. Further results demonstrated that MAFG-DT increased FZD4/5 expression by targeting miR-24-3p, which activated the Wnt/β-catenin pathway to enhance the ability of cell proliferation, migration, invasion and bone metastasis in PCa cells. This study has demonstrated that MAFG-DT may serve as a potential therapeutic target against metastatic PCa.

The Wnt/β-catenin pathway has been shown to participate in the initiation and development of diverse tumor types, including PCa (41–43). CircABCC4, modified by METTL3, was found to promote stemness and PCa metastasis by recruiting IGF2BP2 protein to stabilize CCAR1 mRNA, which activated the Wnt/β-catenin pathway (44). The long transcript of TMPO-AS1 (TMPO-AS1_L_) increased PCa cell invasion, proliferation, and epithelial-to-mesenchymal transition (EMT) through the regulation of the CSNK2A1/DDX3X/Wnt/β-catenin signaling axis (45). The demethylation-induced upregulation of GIPC2 also promoted metastasis of PCa via directly interacting FZD7, further activating the Wnt/β-catenin signaling (46). FZD4/5 are the key component proteins of wnt/β-catenin signaling, which play oncogenic roles in a variety of tumor types. FZD4 has been demonstrated to regulate LINC00319-induced proliferation and metastasis in oral squamous cell carcinoma (47). The translation of FZD5 mRNA was promoted by YTHDF1 in an m^6^A-dependent manner, which subsequently facilitated liver cancer progression (48, 49). In this study, FZD4/5 was found to mediate MAFG-DT-induced oncogenic functions in PCa.

## Conclusions

In conclusion, the present study has provided novel insights into the molecular mechanism through which MAFG-DT promotes PCa cell proliferation, invasion, migration, and bone metastasis, suggesting that MAFG-DT may serve as a promising therapeutic target against PCa bone metastasis.

## Data Availability

The raw data supporting the conclusions of this article will be made available by the authors, without undue reservation.

## References

[1] Lang C Dai Y Wu Z Yang Q He S Zhang X. SMAD3/SP1 complex-mediated constitutive active loop between lncRNA PCAT7 and TGF-β signaling promotes prostate cancer bone metastasis. Mol Oncol. (2020) 14:808–28. doi: 10.1002/1878-0261.12634 PMC7138406 31925912

[2] Yang Q Lang C Wu Z Dai Y He S Guo W. MAZ promotes prostate cancer bone metastasis through transcriptionally activating the KRas-dependent RalGEFs pathway. J Exp Clin Cancer Res. (2019) 38:391. doi: 10.1186/s13046-019-1374-x 31488180 PMC6729064

[3] Lang C Yin C Lin K Li Y Yang Q Wu Z. m(6) A modification of lncRNA PCAT6 promotes bone metastasis in prostate cancer through IGF2BP2-mediated IGF1R mRNA stabilization. Clin Transl Med. (2021) 11:e426. doi: 10.1002/ctm2.v11.6 34185427 PMC8181202

[4] Dai YH Wu ZQ Lang CD Zhang X He SF Yang Q. Copy number gain of ZEB1 mediates a double-negative feedback loop with miR-33a-5p that regulates EMT and bone metastasis of prostate cancer dependent on TGF-beta signaling. Theranostics. (2019) 9:6063–79. doi: 10.7150/thno.36735 PMC6735523 31534537

[5] Wu J Sun H Li J Guo Y Zhang K Lang C. Increased survival of patients aged 0-29 years with osteosarcoma: A period analysis 1984-2013. Cancer Med. (2018) 7:3652–61. doi: 10.1002/cam4.2018.7.issue-8 PMC6089162 29992762

[6] Mo C Huang B Zhuang J Jiang S Guo S Mao X. LncRNA nuclear-enriched abundant transcript 1 shuttled by prostate cancer cells-secreted exosomes initiates osteoblastic phenotypes in the bone metastatic microenvironment via miR-205-5p/runt-related transcription factor 2/splicing factor proline- and glutamine-rich/polypyrimidine tract-binding protein 2 axis. Clin Transl Med. (2021) 11:e493. doi: 10.1002/ctm2.v11.8 34459124 PMC8351523

[7] Mattick JS Amaral PP Carninci P Carpenter S Chang HY Chen L-L. Long non-coding RNAs: definitions, functions, challenges and recommendations. Nat Rev Mol Cell Biol. (2023) 24:430–47. doi: 10.1038/s41580-022-00566-8 PMC10213152 36596869

[8] Sun CC Zhu W Li SJ Hu W Zhang J Zhuo Y. FOXC1-mediated LINC00301 facilitates tumor progression and triggers an immune-suppressing microenvironment in non-small cell lung cancer by regulating the HIF1alpha pathway. Genome Med. (2020) 12:77. doi: 10.1186/s13073-020-00773-y 32878637 PMC7466809

[9] Silva-Fisher JM Dang HX White NM Strand MS Krasnick BA Rozycki EB. Long non-coding RNA RAMS11 promotes metastatic colorectal cancer progression. Nat Commun. (2020) 11:2156. doi: 10.1038/s41467-020-15547-8 32358485 PMC7195452

[10] Shi Q Li Y Li S Jin L Lai H Wu Y. LncRNA DILA1 inhibits Cyclin D1 degradation and contributes to tamoxifen resistance in breast cancer. Nat Commun. (2020) 11:5513. doi: 10.1038/s41467-020-19349-w 33139730 PMC7608661

[11] Liu J Xu R Mai SJ Ma YS Zhang MY Cao PS. LncRNA CSMD1-1 promotes the progression of Hepatocellular Carcinoma by activating MYC signaling. Theranostics. (2020) 10:7527–44. doi: 10.7150/thno.45989 PMC7359090 32685003

[12] Dai T Zhang X Zhou X Hu X Huang X Xing F. Long non-coding RNA VAL facilitates PKM2 enzymatic activity to promote glycolysis and Malignancy of gastric cancer. Clin Transl Med. (2022) 12:e1088. doi: 10.1002/ctm2.v12.10 36229913 PMC9561166

[13] Gao Y Zhang N Zeng Z Wu Q Jiang X Li S. LncRNA PCAT1 activates SOX2 and suppresses radioimmune responses via regulating cGAS/STING signalling in non-small cell lung cancer. Clin Transl Med. (2022) 12:e792. doi: 10.1002/ctm2.v12.4 35415876 PMC9005924

[14] Liu Y Li C Fang L Wang L Liu H Tian H. Lipid metabolism-related lncRNA SLC25A21-AS1 promotes the progression of oesophageal squamous cell carcinoma by regulating the NPM1/c-Myc axis and SLC25A21 expression. Clin Transl Med. (2022) 12:e944. doi: 10.1002/ctm2.v12.6 35735113 PMC9218933

[15] Luo Y Huang S Wei J Zhou H Wang W Yang J. Long noncoding RNA LINC01606 protects colon cancer cells from ferroptotic cell death and promotes stemness by SCD1-Wnt/β-catenin-TFE3 feedback loop signalling. Clin Transl Med. (2022) 12:e752. doi: 10.1002/ctm2.v12.4 35485210 PMC9052012

[16] Zhu Y Zhou B Hu X Ying S Zhou Q Xu W. LncRNA LINC00942 promotes chemoresistance in gastric cancer by suppressing MSI2 degradation to enhance c-Myc mRNA stability. Clin Transl Med. (2022) 12:e703. doi: 10.1002/ctm2.v12.1 35073459 PMC8785984

[17] Ye L Feng W Weng H Yuan C Liu J Wang Z. MAFG-AS1 aggravates the progression of pancreatic cancer by sponging miR-3196 to boost NFIX. Cancer Cell Int. (2020) 20:591. doi: 10.1186/s12935-020-01669-y 33298078 PMC7724861

[18] Ruan Z Deng H Liang M Xu Z Lai M Ren H. Downregulation of long non-coding RNA MAFG-AS1 represses tumorigenesis of colorectal cancer cells through the microRNA-149-3p-dependent inhibition of HOXB8. Cancer Cell Int. (2020) 20:511. doi: 10.1186/s12935-020-01485-4 33093810 PMC7574567

[19] Li D Zhong S Zhu Z Jiang X Zhang J Gu J. LncRNA MAFG-AS1 promotes the progression of bladder cancer by targeting the miR-143-3p/COX-2 axis. Pathobiology. (2020) 87:345–55. doi: 10.1159/000509957 33238264

[20] Li C Wu R Xing Y. MAFG-AS1 is a novel clinical biomarker for clinical progression and unfavorable prognosis in gastric cancer. Cell Cycle. (2020) 19:601–9. doi: 10.1080/15384101.2020.1728017 PMC7100986 32079456

[21] Schaefer KN Peifer M. Wnt/beta-catenin signaling regulation and a role for biomolecular condensates. Dev Cell. (2019) 48:429–44. doi: 10.1016/j.devcel.2019.01.025 PMC6386181 30782412

[22] Farooqi AA de la Roche M Djamgoz MBA Siddik ZH. Overview of the oncogenic signaling pathways in colorectal cancer: Mechanistic insights. Semin Cancer Biol. (2019) 58:65–79. doi: 10.1016/j.semcancer.2019.01.001 30633978

[23] Li Q Fan J Zhou Z Ma Z Che Z Wu Y. AID-induced CXCL12 upregulation enhances castration-resistant prostate cancer cell metastasis by stabilizing β-catenin expression. iScience. (2023) 26:108523. doi: 10.1016/j.isci.2023.108523 38162032 PMC10755053

[24] Han P Li JW Zhang BM Lv JC Li YM Gu XY. The lncRNA CRNDE promotes colorectal cancer cell proliferation and chemoresistance via miR-181a-5p-mediated regulation of Wnt/beta-catenin signaling. Mol Cancer. (2017) 16:9. doi: 10.1186/s12943-017-0583-1 28086904 PMC5237133

[25] Zheng H Chen C Luo Y Yu M He W An M. Tumor-derived exosomal BCYRN1 activates WNT5A/VEGF-C/VEGFR3 feedforward loop to drive lymphatic metastasis of bladder cancer. Clin Transl Med. (2021) 11:e497. doi: 10.1002/ctm2.v11.7 34323412 PMC8288020

[26] Wangzhou K Gong L Liu C Tan Y Chen J Li C. LncRNA MAFG-AS1 regulates human periodontal ligament stem cell proliferation and Toll-like receptor 4 expression. Oral Dis. (2020) 26:1302–7. doi: 10.1111/odi.13330 32176822

[27] Qu Y Liu J. lncRNA MAFG-AS1 Contributes to Esophageal Squamous-Cell Carcinoma Progression via Regulating miR143/LASP1. Onco Targets Ther. (2020) 13:8359–70. doi: 10.2147/OTT.S258157 PMC7445532 32903907

[28] Campbell JP Merkel AR Masood-Campbell SK Elefteriou F Sterling JA. Models of bone metastasis. J Vis Exp. (2012) 67:e4260. doi: 10.3791/4260-v PMC3490264 22972196

[29] Canzio D Nwakeze CL Horta A Rajkumar SM Coffey EL Duffy EE. Antisense lncRNA transcription mediates DNA demethylation to drive stochastic protocadherin α Promoter choice. Cell. (2019) 177:639–653.e15. doi: 10.1016/j.cell.2019.03.008 30955885 PMC6823843

[30] Zhuang J Shen L Yang L Huang X Lu Q Cui Y. TGFbeta1 promotes gemcitabine resistance through regulating the lncRNA-LET/NF90/miR-145 signaling axis in bladder cancer. Theranostics. (2017) 7:3053–67. doi: 10.7150/thno.19542 PMC5566105 28839463

[31] Yin D Hu ZQ Luo CB Wang XY Xin HY Sun RQ. LINC01133 promotes hepatocellular carcinoma progression by sponging miR-199a-5p and activating annexin A2. Clin Transl Med. (2021) 11:e409. doi: 10.1002/ctm2.v11.5 34047479 PMC8101537

[32] Zhou Q Guo J Huang W Yu X Xu C Long X. Linc-ROR promotes the progression of breast cancer and decreases the sensitivity to rapamycin through miR-194-3p targeting MECP2. Mol Oncol. (2020) 14:2231–50. doi: 10.1002/1878-0261.12700 PMC7463371 32335998

[33] Zhang X Bai J Yin H Long L Zheng Z Wang Q. Exosomal miR-1255b-5p targets human telomerase reverse transcriptase in colorectal cancer cells to suppress epithelial-to-mesenchymal transition. Mol Oncol. (2020) 14:2589–608. doi: 10.1002/1878-0261.12765 PMC7530775 32679610

[34] Zhang J Jiang M Qian L Lin X Song W Gao Y. The STAT3-miR-223-TGFBR3/HMGCS1 axis modulates the progression of cervical carcinoma. Mol Oncol. (2020) 14:2313–31. doi: 10.1002/1878-0261.12737 PMC7463355 32491253

[35] Miele E Po A Mastronuzzi A Carai A Besharat ZM Pediconi N. Downregulation of miR-326 and its host gene β-arrestin1 induces pro survival activity of E2F1 and promotes medulloblastoma growth. Mol Oncol. (2020) 15:523–42. doi: 10.1002/1878-0261.12800 PMC7858128 32920979

[36] Massillo C Duca RB Lacunza E Dalton GN Farré PL Taha N. Adipose tissue from metabolic syndrome mice induces an aberrant miRNA signature highly relevant in prostate cancer development. Mol Oncol. (2020) 14:2868–83. doi: 10.1002/1878-0261.12788 PMC7607170 32875710

[37] Liu W Liu P Gao H Wang X Yan M. Long non-coding RNA PGM5-AS1 promotes epithelial-mesenchymal transition, invasion and metastasis of osteosarcoma cells by impairing miR-140-5p-mediated FBN1 inhibition. Mol Oncol. (2020) 14:2660–77. doi: 10.1002/1878-0261.12711 PMC7530781 32412676

[38] Li YJ Yang Z Wang YY Wang Y. Long noncoding RNA ZNF667-AS1 reduces tumor invasion and metastasis in cervical cancer by counteracting microRNA-93-3p-dependent PEG3 downregulation. Mol Oncol. (2019) 13:2375–92. doi: 10.1002/1878-0261.12565 PMC6822248 31420931

[39] Tian X Wu Y Yang Y Wang J Niu M Gao S. Long noncoding RNA LINC00662 promotes M2 macrophage polarization and hepatocellular carcinoma progression via activating Wnt/β-catenin signaling. Mol Oncol. (2020) 14:462–83. doi: 10.1002/1878-0261.12606 PMC6998656 31785055

[40] Qian CJ Xu ZR Chen LY Wang YC Yao J. LncRNA MAFG-AS1 Accelerates Cell Migration, Invasion and Aerobic Glycolysis of Esophageal Squamous Cell Carcinoma Cells via miR-765/PDX1 Axis. Cancer Manag Res. (2020) 12:6895–908. doi: 10.2147/CMAR.S262075 PMC7415466 32801913

[41] Wang X Qian T Bao S Zhao H Chen H Xing Z. Circulating exosomal miR-363-5p inhibits lymph node metastasis by downregulating PDGFB and serves as a potential noninvasive biomarker for breast cancer. Mol Oncol. (2021) 15:2466–79. doi: 10.1002/1878-0261.13029 PMC8410538 34058065

[42] Vava A Paccez JD Wang Y Gu X Bhasin MK Myers M. DCUN1D1 is an essential regulator of prostate cancer proliferation and tumour growth that acts through neddylation of cullin 1, 3, 4A and 5 and deregulation of wnt/catenin pathway. Cells. (2023) 12:1973. doi: 10.3390/cells12151973 37566052 PMC10417424

[43] Liao S Fang X Zhou K Zhao T Ji L Zhang W. LINC00482 sponged miR-2467-3p to promote bone metastasis of prostate cancer through activating Wnt/β-catenin signaling pathway. J Bone Oncol. (2023) 41:100494. doi: 10.1016/j.jbo.2023.100494 37575527 PMC10413070

[44] Huang C Xu R Zhu X Jiang H. m6A-modified circABCC4 promotes stemness and metastasis of prostate cancer by recruiting IGF2BP2 to increase stability of CCAR1. Cancer Gene Ther. (2023) 30:1426–40. doi: 10.1038/s41417-023-00650-x 37563361

[45] Wang M Yin C Wu Z Wang X Lin Q Jiang X. The long transcript of lncRNA TMPO-AS1 promotes bone metastases of prostate cancer by regulating the CSNK2A1/DDX3X complex in Wnt/β-catenin signaling. Cell Death Discovery. (2023) 9:287. doi: 10.1038/s41420-023-01585-w 37542040 PMC10403548

[46] Wang L Wang J Yin X Guan X Li Y Xin C. GIPC2 interacts with Fzd7 to promote prostate cancer metastasis by activating WNT signaling. Oncogene. (2022) 41:2609–23. doi: 10.1038/s41388-022-02255-4 PMC9054671 35347223

[47] Jiang X Liu J Li S JIA B Huang Z Shen J. CCL18-induced LINC00319 promotes proliferation and metastasis in oral squamous cell carcinoma via the miR-199a-5p/FZD4 axis. Cell Death Dis. (2020) 11:777. doi: 10.1038/s41419-020-02978-w 32948745 PMC7501282

[48] Liu X Qin J Gao T Li C He B Pan B. YTHDF1 Facilitates the Progression of Hepatocellular Carcinoma by Promoting FZD5 mRNA Translation in an m6A-Dependent Manner. Mol Ther Nucleic Acids. (2020) 22:750–65. doi: 10.1016/j.omtn.2020.09.036 PMC7595883 33230473

[49] Jing ZF Bi JB Li Z Liu X Li J Zhu Y. Inhibition of miR-34a-5p can rescue disruption of the p53-DAPK axis to suppress progression of clear cell renal cell carcinoma. Mol Oncol. (2019) 13:2079–97. doi: 10.1002/1878-0261.12545 PMC6763763 31294899

