# Supplementary material for: MAFG-DT promotes prostate cancer bone metastasis through activation of the Wnt/β-catenin pathway
Source: Front Oncol. 2024 Dec 13;14:1461546. doi: 10.3389/fonc.2024.1461546 (PMC11671513; doi:10.3389/fonc.2024.1461546)
Supplement: Supplementary file 1 [file DataSheet1.docx]

**Supplementary methods**

**Cell lines and culture**

The human prostate cancer cell lines, PC-3, C4-2B, 22RV1, DU145, VCaP and LNCaP and the normal prostate cell line RWPE-1 were used in this study. All cell lines were cultured with the recommended medium supplemented with 10% fetal bovine serum (Life Technologies, USA), streptomycin (100 mg/ml), and penicillin G (100 U/ml) in a humidified atmosphere of 5% CO_2_ at 37 °C.

**Bioinformatics analysis**

*Gene Set Enrichment Analysis (GSEA)* was used in this study to determine whether a defined set of genes show significant, concordant differences between two sample groups divided by the median of MAFG-DT expression in TCGA, MAFG-DT-High (MAFG-DT-H) and MAFG-DT-Low (MAFG-DT-L) groups. GSEA was performed using GSEA 2.2.1 (http://www.broadinstitute.org/gsea) and gene set was obtained from the Molecular Signatures Database v5.2 (http://software.broadinstitute.org/gsea/msigdb). mRNA expression profiles from 499 PCa patients in TCGA were used as input.

LncRNA expression profiles (TPM format) of all 499 prostate cancer tissues and 52 adjacent normal tissues were download form The Cancer Genome Atlas (TCGA) (https://gdc.cancer.gov/). Gene expression was presented as the mean value of multiple probes for each gene after log_2_ (value +1) transformation.

Starbase (https://starbase.sysu.edu.cn/) and Lncbase (https://diana.e-ce.uth.gr/lncbasev3) were used to predict the miRNA binding to MAFG-DT. Cistrome DB (http://cistrome.org/db/#/) and JSPAR (https://jaspar.genereg.net/) were used to analyze transcriptional factors binding to MAFG-DT promoter.

**RNA immunoprecipitation (RIP)**

A RIP assay was performed to investigate the interactions between RNA and protein [1]. The RIP assay was conducted using a ThermoFisher RIP kit (ThermoFisher Scientific, MA, USA) following the manufacturer’s instructions. In brief, the cells were lysed in a RIP lysis buffer, and RNA magnetic beads were conjugated with a human anti-Ago2 antibody or with a negative control normal mouse anti-IgG. Subsequently, the retrieved RNA was assayed using real-time PCR.

**Chromatin immunoprecipitation (ChIP) assay**

ChIP assay was performed as previously described [2]. Cells (1 × 10^7^) were cultured in a 10-cm dish, and 1% formaldehyde solution in order was added to cross-link proteins to DNA. Then, the cells were harvested in 1 ml of SDS lysis buffer supplemented with 5 μl of Protease Inhibitor mixture. Cell lysates were Sonicated cells to shear DNA to fragments of 200 to 1000 bp. An equal aliquot of chromatin supernatants was incubated with anti-Flag (MAFG labeled with Flag) antibody (Sigma), or an anti-IgG antibody (Millipore) overnight at 4 °C with rotation. Immunoprecipitation of the DNA–protein complexes were conducted with 60 μl of Protein G Agarose for 1 h at 4 °C. After reversing the cross-linking of protein–DNA complexes to liberate the DNA, the human MAFG-DT promoter was amplified by real-time PCR.

**Transwell assay**

Migration and invasion assays were performed using a Transwell chamber consisting of 8-mm membrane filter inserts (Corning, NY, USA) with or without Matrigel (BD Biosciences, Franklin Lakes, NJ, USA) coating. PCa cells (2 × 10^4^) were seeded into the upper compartment of Transwell chambers, and the lower chamber of the Transwell was filled with complete media supplemented with 10% FBS. After incubation for 24h, cells migrating or invading to the bottom side of the chamber were fixed with methanol and stained with crystal violet.

**Wound-healing assay**

Cells were seeded in growth medium in 6-well plates. After confirming that the cells had grown and reached 90% confluence, the cells were then wounded by generating scratches with a sterile pipette tip. The wound area was observed at 0 h and 24 h and the migration rate was measured.

**Western blotting**

Total protein was extracted using radioimmunoprecipitation assay buffer (cat. no. 20–188, Sigma; USA). After separating 20 µg total protein using 12% SDS-PAGE, the protein was transferred to PVDF membranes for blocking for 3 h using 5% nonfat milk. Next, the blocked membranes were washed with TBST and reacted with primary antibodies overnight at 4°C. Subsequently, the membranes were incubated with HRP-linked rabbit (1:5000, ab6721, Abcam, USA) or mouse (1:10000, ab6789, Abcam, USA) antibodies for 1 h at 25°C. The membranes were incubated with SuperEnhanced chemiluminescence detection reagent (Applygen, China), and the protein blot was covered with plastic wrap to expose the X-ray film.

**Luciferase reporter assay**

The luciferase reporter assay was performed with the Dual-Luciferase Reporter Assay System (Promega) according to the manufacturer’s protocol. The pmiGLO-based luciferase vector fused or not fused to the wild-type or mutated FZD4, FZD5 or MAFG-DT were transfected into cells using the Lipofectamine 3000 reagent (Invitrogen). Twenty-four hours after transfection, luciferase and Renilla signals were measured using the Dual Luciferase Reporter Assay Kit (Promega), according to the manufacturer’s instructions.

**Subcellular fractionation assays**

The subcellular fractionation assay was performed precisely as described previously [3]. Cells were treated with hypotonic buffer (25mM Tris–HCl (pH7.4), 1mM MgCl2 and 5mM KCl) for 5 min on ice. NP-40 was then added to the buffer at a final concentration of 0.5%, and the sample was left on ice for another 5 min. The supernatant was collected as the cytosolic fraction after centrifugation at 5000 g for 5 min. Resuspended the pellets in resuspension buffer (20 mM HEPES (pH7.9), 400mM NaCl, 1 mM dithiothreitol, 1 mM EGTA, 1 mM EDTA and 1 mM phenylmethyl sulfonyl fluoride). The nuclear fraction was collected by centrifugation at 12,000 g for 5 min.

**Cell proliferation assay**

Cell Counting Kit-8 (CCK-8, Dojindo Crop, Japan) was used for CKK8 assay. 1×103 cells in 100 μL culture were plated in each well of a 96-well plate and incubated at 37 °C overnight for adherence. Every 24 h for a total of 96 h, each well was added with 10 μL of CCK-8 reagent and cells were further incubated for 1.5 h at 37 °C, the optical density (OD) was measured at 450 nm using a microplate reader (Bio-Tek Instruments, USA). For the colony formation assay, cells (1×103 cells per well) were seeded in six-well plates and maintained in medium supplemented with 10% FBS for 10 days. The colonies were then fixed using paraformaldehyde and stained with 0.1% crystal violet; the number of clones that contained more than 50 cells was counted and imaged. EdU assay was performed using EdU Kits (RiboBio, Guangzhou, China) following the manufacturer’s instructions.

**Statistical analysis**

Data analysis was conducted using GraphPad Prism 7.0. Student's t-test (paired or unpaired) was used to determine the differences between the two groups. One-way ANOVA was used for comparisons among multiple groups and Bonferroni test was used following ANOVA for multiple comparisons. Spearman bivariate correlate analysis was used to detect the correlation between two variables. The log-rank test was used to performed survival analysis. The results are shown as the mean±standard deviation, and P < 0.05 was considered to indicate a statistically significant difference.

[1] R.M. Xia, T. Liu, W.G. Li, X.Q. Xu, RNA-binding protein RBM24 represses colorectal tumourigenesis by stabilising PTEN mRNA, Clin Transl Med 11 (2021) e383. CTM2383 [pii]

10.1002/ctm2.383 [doi].

[2] C. Lang, C. Yin, K. Lin, Y. Li, Q. Yang, Z. Wu, H. Du, D. Ren, Y. Dai, X. Peng, m(6) A modification of lncRNA PCAT6 promotes bone metastasis in prostate cancer through IGF2BP2-mediated IGF1R mRNA stabilization, Clin Transl Med 11 (2021) e426. CTM2426 [pii]

10.1002/ctm2.426 [doi].

[3] Y. Wu, Y. Wang, H. Yao, H. Li, F. Meng, Q. Li, X. Lin, L. Liu, MNX1-AS1, a c-Myc induced lncRNA, promotes the Warburg effect by regulating PKM2 nuclear translocation, J Exp Clin Cancer Res 41 (2022) 337. 10.1186/s13046-022-02547-3.

**Supplemental figure**

**
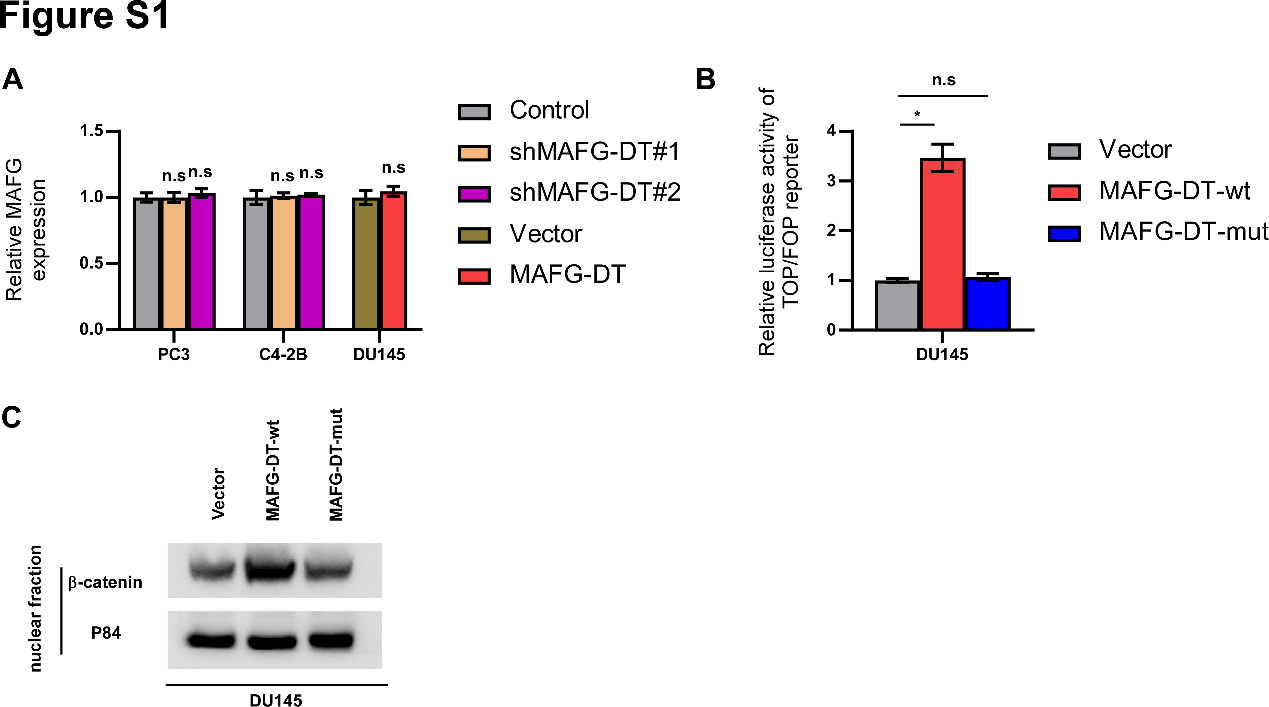
**

**Figure S1. The effect of MAFG-DT on MAFG expression and Wnt/β-catenin signaling.** (A) RT-qPCR assay was performed to determine the MAFG expression in the indicated cells. (B) Luciferase reporter assay was performed to determine the activity of TOP/FOP reporter in the indicated cells. (C) Western blotting assay was performed to determine the protein level of nuclear β-catenin in the indicated cells.

**Supplemental Tables**

**Supplementary Table 1. The sequences of shRNAs and siRNAs.**

| **shRNA Sequence (5’-3’)** | |
| --- | --- |
| shCtrl | AATTCTCCGAACGTGTCACGT |
| shMAFG-DT#1 | GGGCAATTCCAACCAAGAAAC |
| shMAFG-DT#2 | GGAGACTTCGCTGCTTGTTGG |
|  |  |
| **siRNA** | **Sequence (5’-3’)** |
| siCtrl | AATTCTCCGAACGTGTCACGT |
| siMAFG | CCAGCGTCATCACAATAGTAA |
| siZNF777 | GCTGTTAATTTCCTTGACAAT |
| siLMO2 | GCGGGTGAAAGACAAAGTGTA |
| siNR2C2 | CGTCACATTTAAGCTAACAAT |
| siEP300 | CAATTCCGAGACATCTTGAGA |
|  |  |

**Supplementary Table 2. List of primers used for RT-qPCR.**

| **Primer Sequence (5’-3’)** | |
| --- | --- |
| MAFG-DT-F | CTCGGGAGGAAGATAAACGG |
| MAFG-DT-R | CTCACTGACCACGGAACACC |
| FZD4-F | TTCACACCGCTCATCCAGTACG |
| FZD4-R | ACGGGTTCACAGCGTCTCTTGA |
| FZD5-F | TGGAACGCTTCCGCTATCCTGA |
| FZD5-R | GGTCTCGTAGTGGATGTGGTTG |
| U6-F | CTCGCTTCGGCAGCACA |
| U6-R | AACGCTTCACGAATTTGCGT |
| GAPDH-F | GTCTCCTCTGACTTCAACAGCG |
| GAPDH-R | ACCACCCTGTTGCTGTAGCCAA |
|  |  |
|  |  |
